# Classification of genomic components and prediction of genes of *Begomovirus* based on subsequence natural vector and support vector machine

**DOI:** 10.7717/peerj.9625

**Published:** 2020-08-03

**Authors:** Shaojun Pei, Rui Dong, Yiming Bao, Rong Lucy He, Stephen S.-T. Yau

**Affiliations:** 1 Department of Mathematical Sciences, Tsinghua University, Beijing, China; 2 National Genomics Data Center & CAS Key Laboratory of Genome Sciences and Information, Beijing Institute of Genomics, Chinese Academy of Sciences, and China National Center for Bioinformation, Beijing, China; 3 University of Chinese Academy of Sciences, Beijing, China; 4 Department of Biological Sciences, Chicago State University, Chicago, United States of America

**Keywords:** Begomovirus, Classification, Support vector machine, Recursive feature elimination, Subsequence natural vector

## Abstract

**Background:**

Begomoviruses are widely distributed and causing devastating diseases in many crops. According to the number of genomic components, a begomovirus is known as either monopartite or bipartite begomovirus. Both the monopartite and bipartite begomoviruses have the DNA-A component which encodes all essential proteins for virus functions, while the bipartite begomoviruses still contain the DNA-B component. The satellite molecules, known as betasatellites, alphasatellites or deltasatellites, sometimes exist in the begomoviruses. So, the genomic components of begomoviruses are complex and varied. Different genomic components have different gene structures and functions. Classifying the components of begomoviruses is important for studying the virus origin and pathogenic mechanism.

**Methods:**

We propose a model combining Subsequence Natural Vector (SNV) method with Support Vector Machine (SVM) algorithm, to classify the genomic components of begomoviruses and predict the genes of begomoviruses. First, the genome sequence is represented as a vector numerically by the SNV method. Then SVM is applied on the datasets to build the classification model. At last, recursive feature elimination (RFE) is used to select essential features of the subsequence natural vectors based on the importance of features.

**Results:**

In the investigation, DNA-A, DNA-B, and different satellite DNAs are selected to build the model. To evaluate our model, the homology-based method BLAST and two machine learning algorithms Random Forest and Naive Bayes method are used to compare with our model. According to the results, our classification model can classify DNA-A, DNA-B, and different satellites with high accuracy. Especially, we can distinguish whether a DNA-A component is from a monopartite or a bipartite begomovirus. Then, based on the results of classification, we can also predict the genes of different genomic components. According to the selected features, we find that the content of four nucleotides in the second and tenth segments (approximately 150-350 bp and 1,450–1,650 bp) are the most different between DNA-A components of monopartite and bipartite begomoviruses, which may be related to the pre-coat protein (AV2) and the transcriptional activator protein (AC2) genes. Our results advance the understanding of the unique structures of the genomic components of begomoviruses.

## Introduction

The family *Geminiviridae* contains plants viruses of circular single-stranded (ss) DNA with a twinned quasi-icosahedral (geminate) shape. According to the Tenth Report of the International Committee on Taxonomy of Virus (ICTV) published in 2017, the *Geminiviridae* is divided into nine genera: *Begomovirus, Becurtovirus, Mastrevirus, Curtovirus, Capulavirus, Eragrovirus, Turncurtovirus, Topocuvirus* and *Grablovirus (Varsani et al., 2014; Varsani et al., 2017; Zerbini et al., 2017)*. Among these nine genera, the genus *Begomovirus* is the largest geminivirus genus, including more than 420 species *(Dominguez-Duran et al., 2018; Fauquet et al., 2003; Martinez-Marrero et al., 2020)*. Viruses in the other eight genera only have monopartite genomes, whereas those in the genus *Begomovirus* have monopartite or bipartite genomes *(Zerbini et al., 2017)*. The genomes of bipartite begomoviruses consist of two components, referred to as DNA-A and DNA-B, each of 2.5–2.6 kb. The DNA-A component encodes all virus functions required for DNA replication, gene expression, and insect transmission, while DNA-B is responsible for systemic infection. The DNA-A component of bipartite begomoviruses encodes six proteins including the coat protein, the replication-associated protein or AC1 protein, the transcriptional activator protein (TrAP) or AC2 protein, the replication enhancer protein (REn) or AC3 protein, the symptom determinant protein or AC4 protein and AC5 protein which is related to the suppression of antiviral defenses based on RNA silencing *(Li et al., 2015; Van Wezel et al., 2002)*. The DNA-B component has a common region of 200 to 250 bp as DNA-A. But it only encompasses two proteins including movement protein or BV1 protein, and nuclear shuttle protein (NSP) or BC1 protein which are involved in inter- and intracellular movement *(Krenz, Jeske & Kleinow, 2012)*. However, the DNA-A component of monopartite begomoviruses encodes one more protein, pre-coat protein (V2) related to movement and transport of viral genome into the plant *(Wang et al., 2018)*.

Three classes of circular DNA satellites have been associated with begomoviruses: betasatellites, alphasatellites and deltasastellites *(Briddon et al., 2018; Fiallo-Olive, Tovar & Navas-Castillo, 2016; Gnanasekaran et al., 2019; Zhou, 2013)*. Betasatellites of approximately 1.3 kb contain a single ORF termed βC1 and are associated with many monopartite begomoviruses, which play an important role in the induction of typical disease symptoms *(Gnanasekaran et al., 2019; Li et al., 2018)*. Alphasatellites are related to the replication-associated protein -encoding components of nanoviruses. They are associated with betasatellites and have no known contributions to the pathogenesis of begomovirus-betasatellite disease complexes *(Briddon et al., 2018)*. Deltasatellites are approximately 0.7 kb and do not encode any proteins *(Fiallo-Olive, Tovar & Navas-Castillo, 2016)*. The number of genomic components and organization of the genomes are the important criteria to establish the taxonomy of *Begomovirus*. For example, New World begomoviruses are mostly bipartite, do not contain an AV2/V2 ORF, and can be associated with alphasatellites. Classifying the genomic components and predicting the gene of begomoviruses is important for studying its origin and pathogenic mechanism *(Briddon et al., 2010; Zhou, 2013)*.

ICTV classifies nine genera in the *Geminiviridae* family based on host range (monocots or dicots), type of vector (leafhoppers, treehoppers, whiteflies, aphids), genome organization (mono- or bipartite), and phylogenetic relationships *(Zerbini et al., 2017)*. With the continuous development of the high-throughput sequencing methods, thousands of complete genomes or partial sequences of geminiviruses are available in the public datasets. Many different computational methods or algorithms are used to classify geminiviruses only based on the genomic information. For example, Silva et al. (2017b) presented a machine learning (ML) classification model, called Fangorn Forest (F2), based on only genomic characteristics to classify genera and genes in the *Geminiviridae* family. All genera of the family *Geminiviridae* could be classified with high accuracy *(Silva et al., 2017b)*. However, these methods cannot classify the genomic components of begomoviruses. Especially, they cannot distinguish whether a DNA-A component is from a monopartite or a bipartite begomovirus.

In this study, to classify the genomic components and predict the genes of begomoviruses, we propose a machine learning model based on the subsequence natural vector (SNV) method and Support Vector Machine (SVM) algorithm. First, the genome sequence is represented as a numerical natural vector *(He et al., 2020)*. Then SVM is applied to the datasets to build the classification model. Benchmark results demonstrate that our method is highly accurate for the classification than BLAST *(Camacho et al., 2009)*, Random Forest, and Naive Bayes methods. Our model can classify different genomic components of begomoviruses and distinguish whether a DNA-A component is from a monopartite or a bipartite begomovirus. Also, based on the importance of features of the vector by recursive feature elimination (RFE) *(Xiaoqiang, Qing & Jingjing, 2013)*, we find that the content of four nucleotides in the second and tenth segments (approximately 150–350 bp and 1,450–1,650 bp) are the most different between DNA-A components of monopartite and bipartite begomoviruses, which may be related to the AV2 and AC2 genes. Based on the results of classification, we can also predict the genes of different genomic components. All the results demonstrate our method performs well on the genomic components classification and the genes prediction of begomoviruses.

## Materials & Methods

### Materials

Genomes of non-begomoviruses and begomoviruses are collected from the National Center for Biotechnology Information (NCBI). We intend to distinguish genome sequences of begomoviruses from other geminiviruses. Non-begomovirus class is composed of eight genera of *Geminiviridae* family. Complete genomes of the Begomovirus family including DNA-A, DNA-B, alphasatellites, betasatellites, and deltasatellites are used in the classification of sequence types of the *Begomovirus* genus. To ensure the accuracy of the data, we validate the genomes on the Geminivirus data warehouse *(Silva et al., 2017a)*. Then we construct the training set and test set according to the ratio of 7: 3.

The complete DNA-A components of monopartite begomoviruses and bipartite begomoviruses are selected to determine whether the DNA-A is from a bipartite or a monopartite begomovirus. The training set contains the monopartite DNA-A sequences and the bipartite DNA-A sequences that have the corresponding DNA-B segments in NCBI. The remaining DNA-A sequences that do not have corresponding DNA-B segments in NCBI are the test dataset. Accordingly, the genes of genomic components in this training dataset are used as training data for gene prediction, and the genes in this test dataset are for prediction.

The data size used in training and testing datasets is shown in Table 1. All the accession numbers of the complete genomes in the training and testing dataset are shown in Files S1 and S2 respectively.

**Table 1:** The data size used in training and testing datasets of the classification tasks.

	Genus classification	Genome components classification	Monopartite or bipartite DNA-A classification
Training dataset	976	491	349
Test dataset	419	212	120

### Classification model

**Figure 1:**
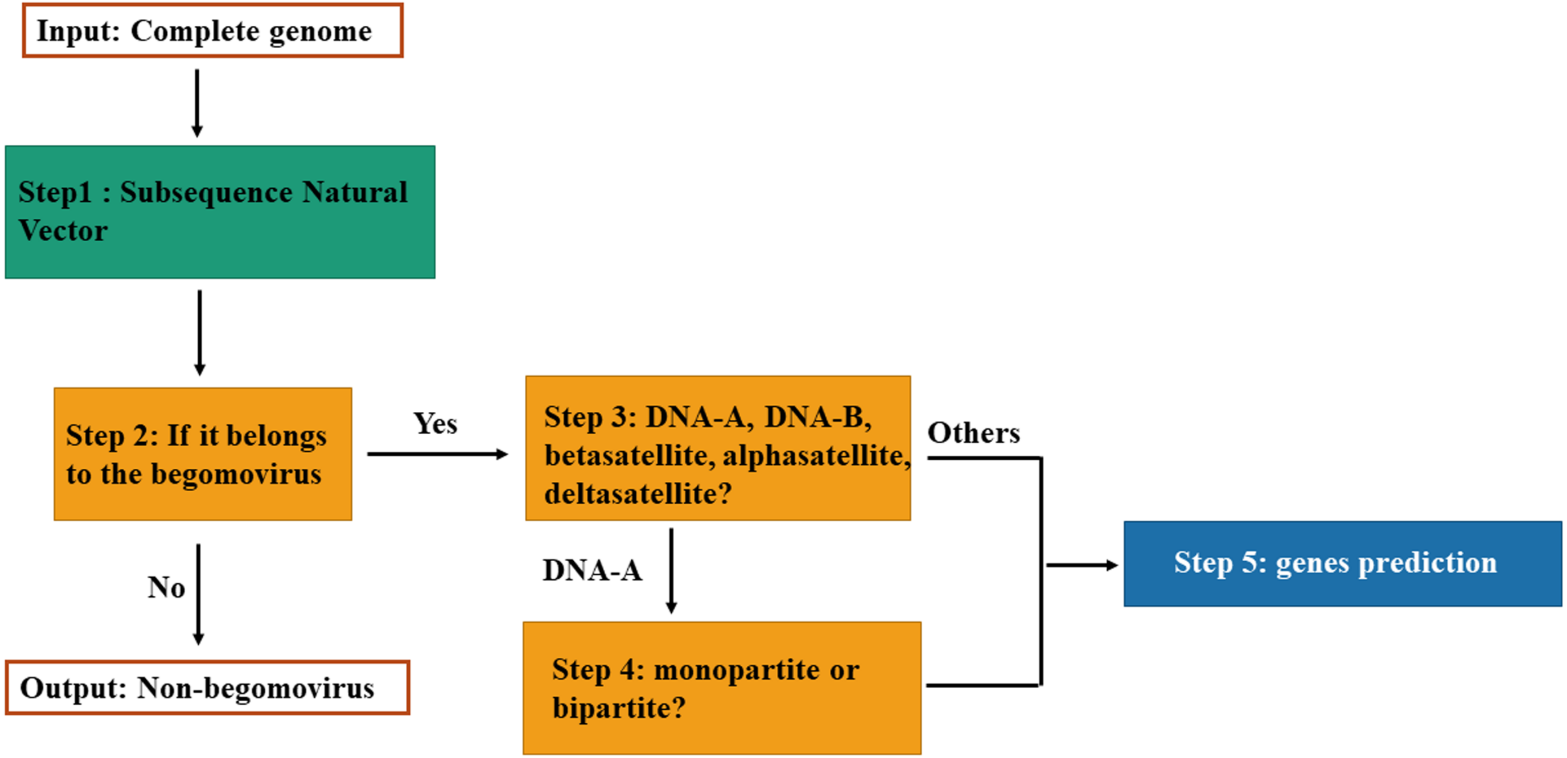
Flowchart of the classification model.

The classification model for genomic components of *Begomovirus* is composed of four fundamental steps as illustrated in Fig. 1. First, we use the SNV method to extract attributes. Each DNA sequence is converted to a vector in the Euclidean space by SNV method. Second, the model can classify the vector as belonging to the *Begomovirus* genus by SVM. Then, if it belongs to the *Begomovirus* genus, it can be classified among DNA-A, DNA-B, betasatellites, alphasatellites, and deltasatellites. And if it is classified as the DNA-A sequence, the model can determine whether it is from a bipartite or monopartite begomovirus. Finally, according to its classification, we can do genes prediction for the input DNA sequence.

#### Attribute extraction: Subsequence Natural Vector (SNV)

To build classification models, the genome sequences are represented numerically first. Let $S={s}_{1}{s}_{2}\ldots {s}_{N},s\in \left\{ A,C,G,T \right\} $ be a DNA sequence of length *N*. To construct Subsequence Natural Vector [17], we fix *k* as a preset integer less than *N*. We divide *N* by *k*:

(1) \begin{eqnarray*}q= \left\lfloor \frac{N}{k} \right\rfloor , r=N-q\cdot k,\end{eqnarray*}

where *q* is the quotient and *r* is the remainder and *k* ∈ {3, 4, 5, 7, 11, 13, 17, 19, 23, 29, 31, 37, 41, 43, 47} (Zhao, He & Yau, 2011). Then the DNA sequence is divided into *k* subsequences: the lengths of the first *r* subsequences are *q* + 1, and the lengths of the remaining *k* − *r* subsequences are *q*.

Next, we define *NV*(*i*) be the Natural Vector of *i*th subsequences as follows:

1. Let *n*_*l*_ be the number of nucleotides *l* in the subsequence.
2. Let $s \left[ l \right] [p]$ be the position of *p*-th nucleotide *l* in the subsequence. Then we take ${\mu }_{l}= \frac{{\mathop{\sum }\nolimits }_{p=1}^{{n}_{l}}s \left[ l \right] [p]}{{n}_{l}} $ as the mean position of nucleotide *l*.
3. We define the second central moments as: ${D}_{2}^{l}= \frac{{\mathop{\sum }\nolimits }_{p=1}^{{n}_{l}}(s \left[ l \right] \left[ p \right] -{\mu }_{l})^{2}}{n{n}_{l}} $, where *n* is the length of the subsequence.
4. $NV \left( i \right) = \left( {n}_{A},{n}_{C},{n}_{G},{n}_{T},{\mu }_{A},{\mu }_{C},{\mu }_{G},{\mu }_{T},{D}_{2}^{A},{D}_{2}^{C},{D}_{2}^{G},{D}_{2}^{T} \right) .$

Finally, we put all $NV \left( i \right) $ together and get Subsequence Natural Vector *SNV* of *S*,

(2) \begin{eqnarray*}SNV=(NV \left( 1 \right) ,\ldots ,NV(k)).\end{eqnarray*}

Then SNV for each sequence is selected as input attributes of SVM algorithm.

#### Evaluation of machine learning methods

In our study, Support Vector Machine (SVM) method is used to perform the classification tasks. SVM is implemented in Classification Learner in MATLAB R2016a with parameters of Preset: medium Gaussian SVM Kernel, function: Gaussian, Kernel scale: 14, Box constraint level: 1, Multiclass method: one-vs-One, Standardize data: true. Other three methods including Random Forest, Naive Bayes and BLAST are tested for comparison. Random Forest is implemented in Classification Learner in MATLAB R2016a with parameters of Preset: Bagged Trees Ensemble, method: Bag, Type: Classification, Number of learners: 30. The function ‘fitcnb’ in MATLAB R2016a is used to train multiclass naive Bayes model with default parameters. Blastn in NCBI-2.10.0+ is used for BLAST analysis. All the codes of our study are shown in File S3.

We use two different techniques to test the performance of the classification tasks. One technique is a completely independent test set, and the other is 10-fold cross-validation. For each test, the following measures are calculated to evaluate the model performance: accuracy, recall, precision, F-measure, and AUC. True positives (TP) are the cases in which the classification model predicts them to be true while those cases were indeed true. True negatives (TN) are corresponding to the cases correctly predicted to be false. False positives (FP) and False negatives (FN) refer to true or false cases that are incorrectly predicted. Accuracy is to measure how often our classification model is correct, calculated as (TP + TN)/ (total number). Recall (R), the proportion of actual negative cases correctly labeled is calculated as TN/ (FP + TN), while precision (P) is the proportion of actual positive cases correctly identified, which is calculated as TP/ (TP + FN). And F-measure is 2*P*R/ (P+R). AUC is defined as the area enclosed by the coordinate axis under the ROC curve.

#### Feature selection

To evaluate the importance of features for the models built on our datasets, we used a MATLAB package SVM-REF (https://www.mathworks.com/matlabcentral/fileexchange/50701-feature-selection-with-svm-rfe, MATLAB Central File Exchange). SVM-RFE is a powerful feature selection algorithm in machine learning *(Xiaoqiang, Qing & Jingjing, 2013)*. The method is suitable to avoid over-fitting when the number of features is high. It is a sequence backward selection algorithm based on SVM maximum interval principle. It uses the model training samples, then ranks each feature, removes the features with the smallest feature score, then trains the model again with the remaining features, performs the next iteration, and finally selects the required number of features.

### Genes prediction model

**Figure 2:**
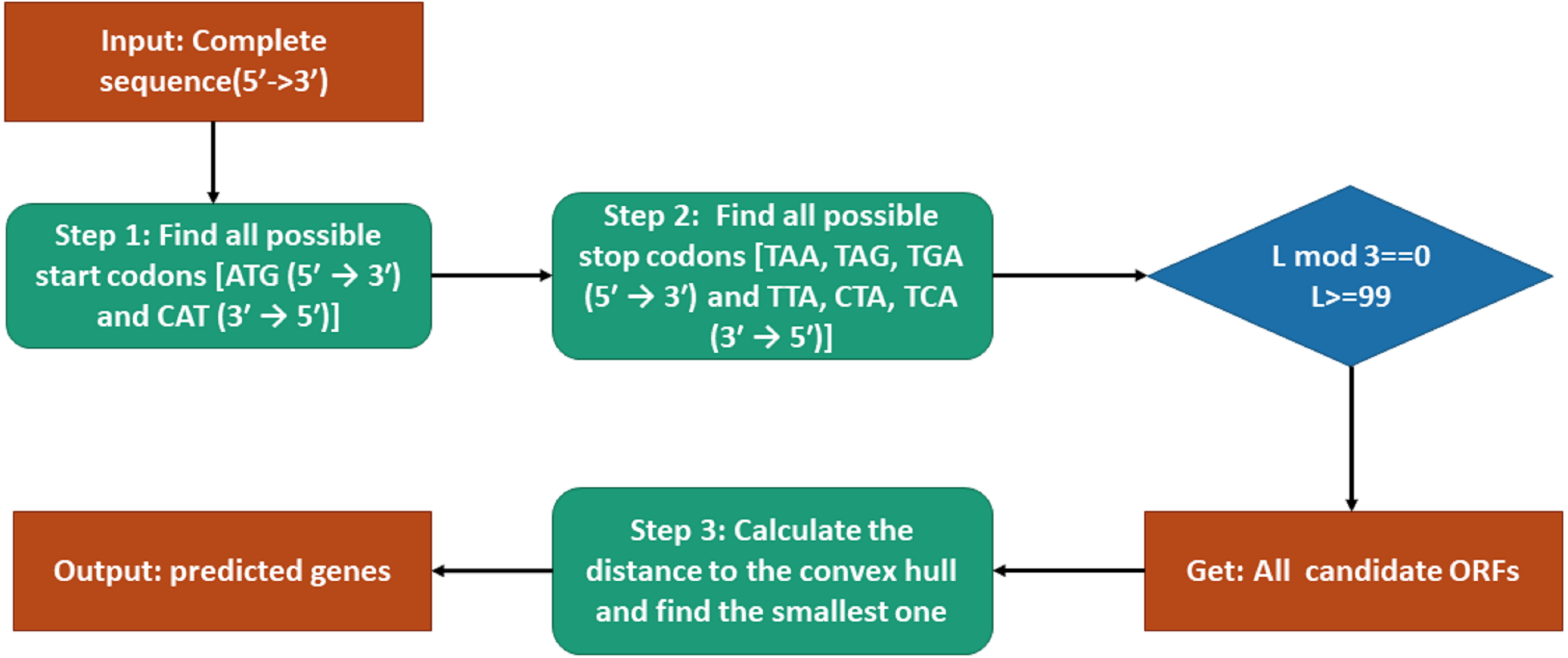
Flowchart of the gene prediction model.

The procedure of genes prediction is shown in Fig. 2. To predict genes by our model, the candidate open reading frames (ORFs) should be selected first. According to that all start codons are ATG (5′ → 3′) and CAT (3′ → 5′), we find all the possible start codons in the sense or anti-sense sequence. Similarly, all stop codons TAG, TAA, TGA TAG, TAA, TGA (5′ → 3′) and CTA, TTA, TCA (3′ → 5′) can be located. Then each fragment between each pair of start codon and stop codon in the same sense is a candidate ORF. However, before starting our model, we need to check the basic requirement of each candidate ORF (in 5′ → 3′ or 3′ → 5′): whether the translated amino acid sequence is not truncated and is greater or equal to 33 amino acids. Then we can obtain all candidate ORFs. The SNVs of different genes of the training data are used to construct convex hulls in Euclidean space. The distances between all the candidate ORFs and convex hulls can be calculated by Tian’s work *(Tian, Zhao & Yau, 2018)*. At last, the closet candidate ORF is our predicted gene.

## Results

### Performance of the classification model

**Figure 3:**
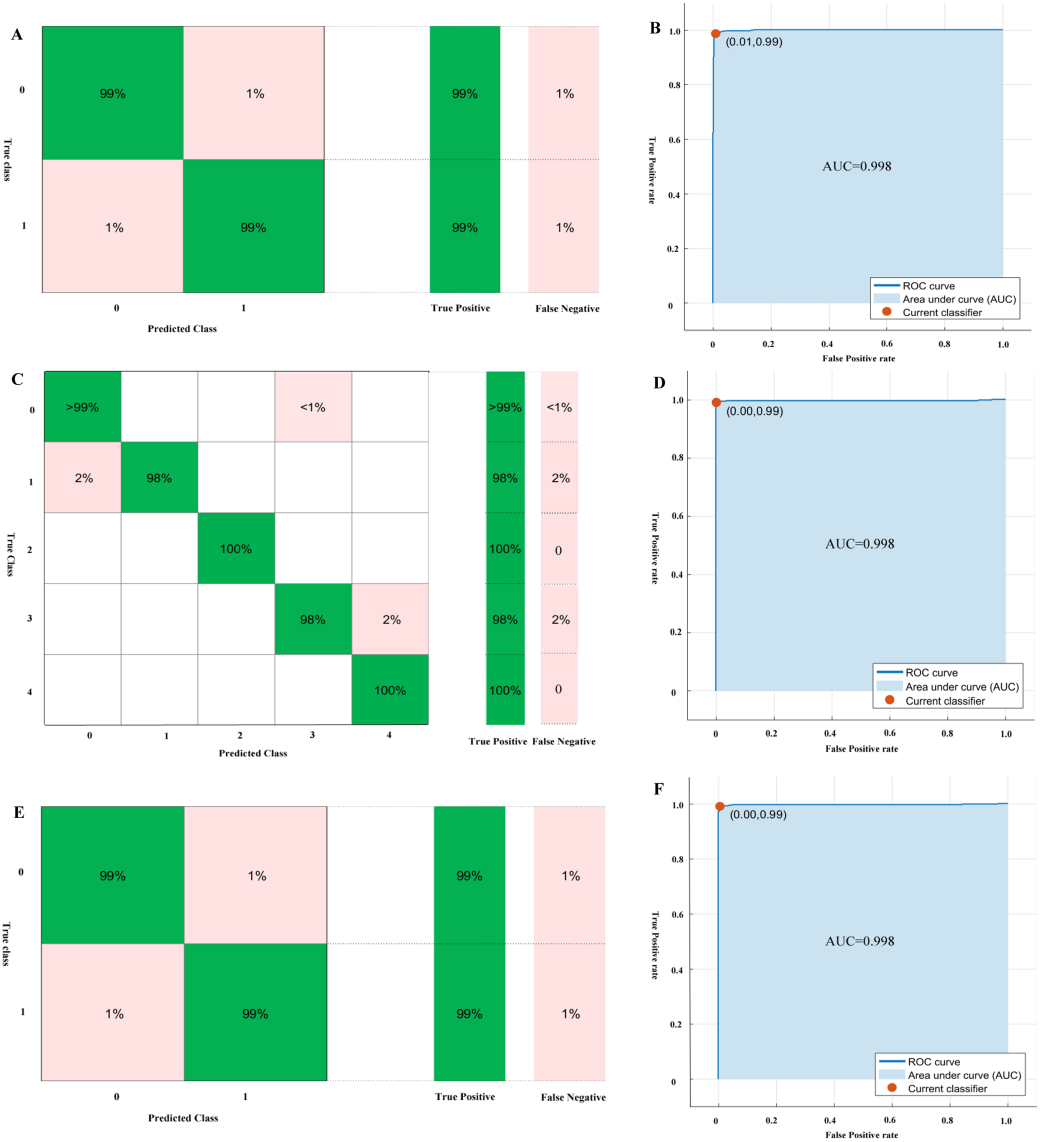
The confusion matrices and ROC curves of genus, genomic components and monopartite or bipartite DNA-A classification by SVM. (A) the confusion matrix of genus classification; (B) the ROC curves of genus classification; (C) the confusion matrix of genomic components classification; (D) the ROC curves of genomic components classification; (E) the confusion matrix of monopartite or bipartite DNA-A classification; (F) the ROC curves of monopartite or bipartite DNA-A classification.

The performance of the models for genus, genomic components, and monopartite or bipartite DNA-A classification by 10-folds validation are shown in Table 2, which are trained by SVM, BLAST, Random Forest and Naive Bayes methods. The performance results show that SVM is superior to other methods for the classification tasks, especially for the monopartite or bipartite DNA-A classification. For the classification of the genus *Begomovirus*, the SVM algorithm achieves the performance of accuracy, recall, precision F-measure and AUC of 0.990. 0.992, 0.987, 0.990, 0.998, respectively. The mean performance of SVM for genomic components classification is the accuracy, recall, precision, F-measure, and AUC of 0.993, 0.983. 0.990, 0.986, 0.998, respectively. For monopartite or bipartite DNA-A classification, the SVM method achieves the accuracy, precision, recall, F-measure, and AUC of 0.987, 0.985, 0.988, 0.990, 0.998, respectively. The confusion matrices and ROC curves of genus, genomic components, and monopartite or bipartite DNA-A classification by SVM are shown in Fig. 3.

**Table 2:** The performance of the classification tasks by SNV-SVM, Random Forest, Naive Bayes and Blastn.

		Accuracy	Precision	Recall	F-measure	AUC	Time (s)
Genus classification	SNV-SVM	0.990	0.987	0.992	0.990	0.998	0.553
	Random Forest	0.980	0.974	0.988	0.981	0.997	3.158
	Naive Bayes	0.867	0.871	0.896	0.884	0.923	1.717
	Blastn	0.990	0.990	0.990	0.990	0.995	5.718
Genome component classification	SNV-SVM	0.995	0.995	0.995	0.995	0.998	1.148
	Random Forest	0.995	0.996	0.996	0.996	0.996	2.765
	Naive Bayes	0.982	0.976	0.988	0.981	0.995	2.050
	Blastn	1.000	1.000	1.000	1.000	1.000	3.745
Mono-or bipartite DNA-A classification	SNV-SVM	0.987	0.985	0.991	0.988	0.998	0.293
	Random Forest	0.952	0.947	0.953	0.952	0.980	1.862
	Naive Bayes	0.977	0.967	0.987	0.977	0.973	0.219
	Blastn	0.818	0.750	0.844	0.794	0.834	1.050

Then we use SVM to predict the test dataset. And the accuracy for genus, genomic components, and monopartite or bipartite DNA-A classification is 0.990, 0.981, and 0.972, respectively. Especially, for monopartite or bipartite DNA-A classification, there are five mismatches. Only one of DNA-As which is predicted as bipartite is inconsistent with corresponding reference (NC013413). The biochemical experiments on a begomovirus DNA-B component or virus-associated satellite DNA cannot find in any of the samples by PCR using the DNA-B general primer pairs DNABLC1/DNABLV2 and DNABLC2/DNABLV2 and the satellite detection primer pair Beta01/Beta02 *(Tsai et al., 2009)*. However, sometimes the DNA-B component (DNA-B) cannot be successfully tested, maybe owning to the low concentration of this component in infected tissues *(Salim & Thushari, 2010)*. For those predicted as monopartite, three bipartite begomoviruses are misclassified (NC014845, NC014894, and NC030403). But the phylogenetic analysis indicates that the three sequences are typical old-world bipartite begomoviruses (Leke et al., 2016; Qazi et al., 2007; Venkataravanappa et al., 2012). As a result, the organization of genes of these three bipartite is closer to monopartite begomoviruses.

### Performance of the gene prediction model

**Figure 4:**
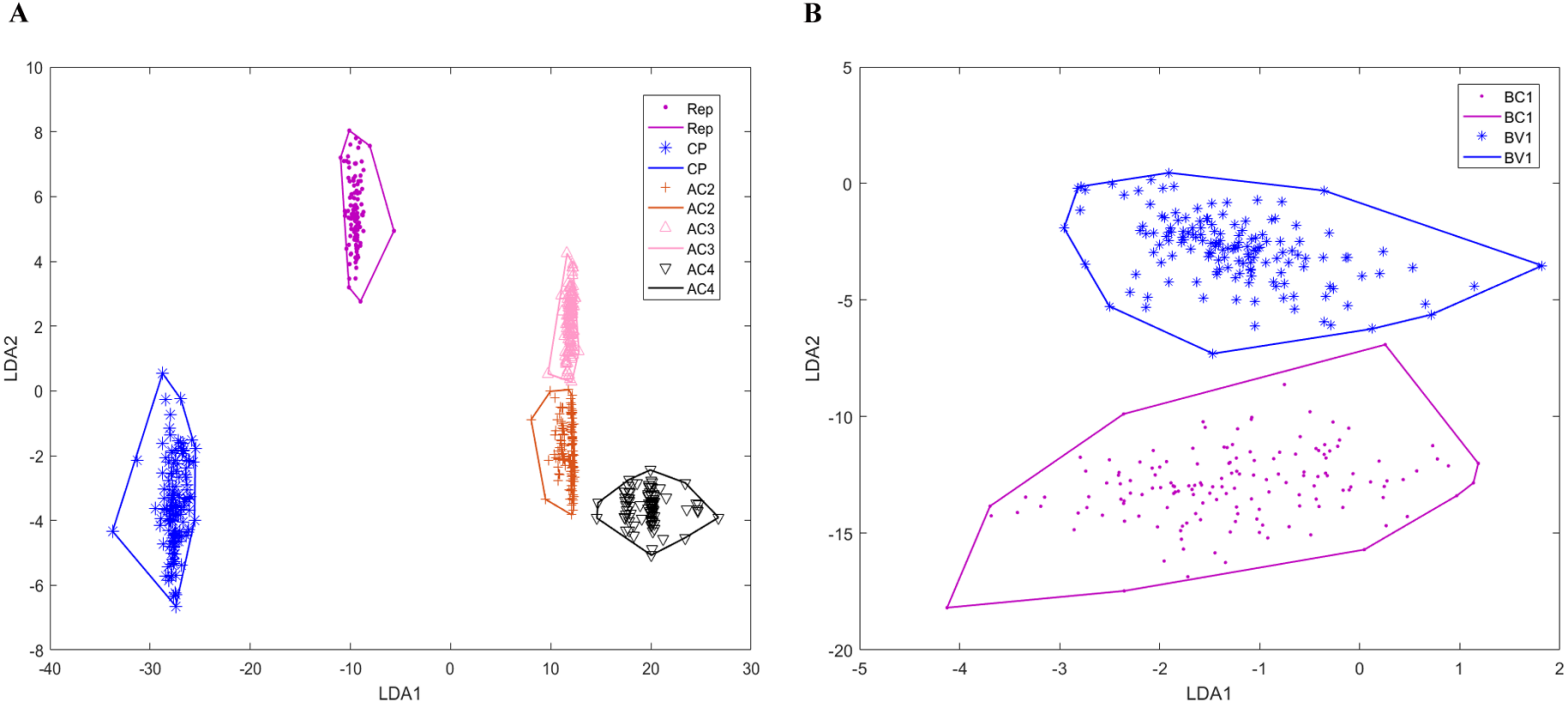
Two-dimensional projection of the convex hulls of different genes of different DNA types by Linear Discrimination Analysis. *X*-axis and *Y*-axis are two directions of projection. (A) Two-dimensional projection of the convex hulls of different genes DNA-A; (B) two-dimensional projection of the convex hulls of different genes DNA-B.

Genomic components have various numbers of genes that encoding different proteins. Bipartite DNA-As contain six ORFs: coat protein (CP), replication-associated protein (Zerbini et al.), AC2, AC3, AC4, and AC5, while monopartite DNA-As have seven ORFs: CP, V2, Rep, C2, C3, C4 and C5. DNA-Bs encode two ORFs: BV1 and BC1. Betasatellites and alphasatellites encode βC1 and Rep proteins, respectively. Because AC5 proteins are recently studied, the number of AC5 proteins is significantly less than other proteins. There are only 15 bipartite DNA-A sequences annotated AC5 protein in our dataset, so the prediction of AC5 protein is ignored in our model. After the classification model established for begomoviruses, we can accurately determine the genomic component of a given sequence of the virus. Then we use training data to construct the convex hulls of the virus genes. Figure 4 is two-dimensional projection of the convex hulls of the genes in genomic components by linear discrimination analysis. We can see that for each genomic component, the convex hulls of genes are disjoint from each other. Because the genomic structures of DNA-B and satellites are relatively simple, all the prediction results of DNA-B and satellites by our model are consistent with gene annotation on NCBI. For DNA-A, there are four Reps and one AC2 predicted wrong which are shorter than annotation on NCBI, and one AV1 predicted longer than annotation. So, the mean accuracy of our prediction model of DNA-A components is 0.9791.

### The importance of features

The sequences of DNA-A components in the monopartite and bipartite begomoviruses are similar, but it can be classified with high accuracy by our model. So, we can further study the differences of DNA-A genome sequences between bipartite and monopartite begomoviruses by the importance of features in the classification model. Because of the best performance of classification by SVM, REF in SVM is applied to perform variable selection. The result of predictor importance by Random Forest is also shown in Table S1. According to the importance of features by RFE, the top eight dimensions of SNV are 109th to 112th dimensions and 13th to 16th dimensions, which are numbers of four nucleotides in the tenth and second segments. It means that the contents of four nucleotides in the tenth and second segments are the most divergent in monopartite and bipartite begomoviruses.

To further study the differences in the tenth and second segments, we calculate the GC content of the second and tenth genome segments of all the DNA-A genome sequences. The GC content between monopartite and bipartite DNA-A components is compared with the independent *T*-test. The results are shown in Table 3. We can conclude that the average GC content of the second segment of the DNA-A genome sequences of bipartite begomoviruses is significantly higher than that of the DNA-A genome sequences of monopartite begomoviruses. However, the average GC content of the tenth segment of the DNA-A genome sequences of bipartite begomoviruses is significantly lower than that of the DNA-A genome sequences of monopartite begomoviruses.

**Table 3:** The results of *T*-test of GC content.

	Monopartite	Bipartite	T-statistic	*p*-value
GC content (2nd segment)	0.430(±0.044)	0.474(±0.047)	−7.578	0.000
GC content (10th segment)	0.493(±0.035)	0.479(±0.037)	2.962	0.003

**Figure 5:**
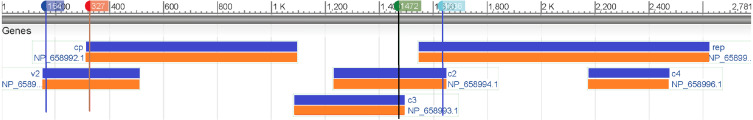
Genomic regions, transcripts, and products of a monopartite begomoviruse (Tomato yellow leaf curl virus, NC_004005).

Then we map these two segments to the original sequence and find that the second segment is located at the V2 (AV2) gene and the other one is located at the C2 (AC2) gene (Fig. 5). The divergence of genes between monopartite and bipartite begomoviruses has been well documented. For example, in *(Matic, Pegoraro & Noris, 2016; Mubin et al., 2010)*, because monopartite begomoviruses do not have a DNA B component and therefore lack the nuclear shuttle protein (NSP) that is the elicitor of cell death, they argued that the protein encoded by the V2 gene of monopartite begomoviruses was a pathogen-encoded a virulence determinant and the cell death elicited by the V2 protein was suppressed by the product encoded by the C2 gene, a homolog of the bipartite begomovirus-encoded TrAP.

## Discussion

This paper proposes a new model based on SNV method and SVM algorithm to classify the genomic components of *Begomovirus*. According to the results, our classification model can classify DNA-A, DNA-B, and different satellites with high accuracy. Especially, it can distinguish whether a DNA-A component is from a monopartite or a bipartite begomovirus. Then we can predict the genes of the sequence based on its genomic components by our model accurately. To further investigate the differences between the DNA-A sequences, we examine the feature importance of the dimensions of SNV by REF algorithm. There is a significant difference in GC content between the second and tenth segments (approximately 150–350 bp and 1,450–1,650 bp) of monopartite and bipartite begomoviruses, which may be related to the AV2 and AC2 genes.

The previous studies only classify the satellites of begomoviruses based on the proportion of nucleotides. In our study, five different genomic components of begomovirueses can be classified accurately based on the proportion and the distribution of four nucleotides. Our method can identify multiple begomovirus sequences at the same time. Particularly when the number of sequences is large, our method has more obvious advantages. So, it is a powerful customized method for the classification of begomoviruses with high accuracy that can result in expressive economic impacts.

## Conclusions

The genus *Begomovirus* is the largest genus of *Geminiviridae,* and comprises viruses with either monopartite or bipartite genomes, and some satellites. In addition, the sequence similarity between DNA-A components of monopartite and bipartite begomoviruses is very high. Identifying DNA components of this genus has become a challenge due to high similarity and many sequences available in databases. In this study, we propose a classification model based on SNV method and SVM algorithm. The SNV method can compare the genome sequences accurately by dividing the sequences into several segments and then extract the proportion and the distribution of for nucleotides of each segment. SVM is applied to build the classification models. The results show that our method is efficient to classify genomic components of begomoviruses and predict genes of begomoviruses with high precision. In addition, each genome sequence is associated with a numerical vector in the Euclidean space by SNV method. Then, the Euclidean distance between different vectors can be used to measure the biology distance between sequences. Therefore, our method can be used to perform the phylogenetic analysis based on the Euclidean distance.

##  Supplemental Information

10.7717/peerj.9625/supp-1 Supplemental Information 1 The accession numbers of sequences of the training dataset of the genus classification, the classification of the genomic components and the classification of the monopartite or bipartite DNA-A classification

10.7717/peerj.9625/supp-2 Supplemental Information 2 The accession numbers of sequences of the test dataset of the genus classification, the classification of the genomic components and the classification of the monopartite or bipartite DNA-A classification

10.7717/peerj.9625/supp-3 Supplemental Information 3 The MATLAB code of the pipeline of the SNV and SWM model main: the main function of the pipeline. SNV_f: the function of the Subsequence Natural Vector. SVM_f: the function of the Support Vector Machine. RF_f: the function of the Random Forest. training_genus.fasta: the training data of the genus classification.

10.7717/peerj.9625/supp-4 Supplemental Information 4 Top 5 on the predictor importance list by Random Forest

## Additional Information and Declarations

### Data Availability

The following information was supplied regarding data availability: The raw data is available in the Supplementary Files.
