# Supplementary material for: Classification of genomic components and prediction of genes of Begomovirus based on subsequence natural vector and support vector machine
Source: PeerJ. 2020 Aug 3;8:e9625. doi: 10.7717/peerj.9625 (PMC7409808; doi:10.7717/peerj.9625)
Supplement: Supplemental Information 4 [file peerj-08-9625-s004.docx]

Table S1: top 5 on the predictor importance list by Random Forest

|  | The value of importance |
| --- | --- |
| Dimension 149 | 0.5097 |
| Dimension 29 | 0.4854 |
| Dimension 110 | 0.456 |
| Dimension 15 | 0.4506 |
| Dimension 77 | 0.4358 |
